# Editorial – ethical practice and genomic research

**DOI:** 10.1080/11287462.2020.1855712

**Published:** 2020-12-09

**Authors:** Janet Seeley, Michael Parker

**Affiliations:** a London School of Hygiene and Tropical Medicine, London, United Kingdom; b Ethox Centre, Nuffield Department of Population Health, University of Oxford, United Kingdom

Genomic research offers the potential of significant improvements in diagnosis, treatment, and in health care more broadly. The achievement of these benefits against a background of well-founded public trust and confidence depends crucially upon the addressing of a range of important ethical challenges such as those related to consent, privacy, ownership of samples and data sharing (Cambon-Thomsen et al., 2007). More recently, the range of such issues has expanded to include a number of questions relating to the feedback of findings, and the appropriate nature of the relationship between genomic research and medical practice. When genomic studies are conducted in the context of international collaborative research and in settings with low literacy levels, such issues arise with even greater intensity, and are compounded by further challenges (Parker & Kwiatkowski, 2016; Ramsay et al., 2014). Some of the key issues highlighted in the global heath ethics literature include how to explain key scientific concepts in lay terms to improve research understanding, the model of consent that is appropriate for future uses of human biological samples, the value and place of community engagement, benefit-sharing, ownership and control of samples, associated data and sample and data sharing, and questions relating to fair collaborations (de Vries et al., 2011; Munung et al., 2016; Nyika, 2009; Parker et al., 2016; Tindana et al., 2012; Upshur et al., 2007; Wonkam et al., 2011).

One area of intense debate in recent times has been the model of consent that should be appropriate for genomic and biobanking studies in low and middle-income countries, and what type of governance mechanism is required for safeguarding the interest of research participants and their communities in such studies. A growing number of empirical studies from Africa have highlighted the challenges with obtaining and documenting consent in lower and middle income countries, particularly in sub-Saharan Africa (Bull et al., 2012; Mandava et al., 2012; Marshall, 2008; Molyneux et al., 2004; Tekola et al., 2009; Tindana et al., 2006). The literature suggests that recent advances in research, such as genomic studies, may have further complicated consent processes due to the complex nature of these studies and unfamiliarity with the concepts and methodologies involved. One recommendation for addressing these challenges is exploring innovative ways of explaining complex scientific terms in lay terms to research participants, including appropriate levels of community engagement (CE) (Tindana et al., 2012).

Several proposals have been made to address the issue of consent in genomic and biobanking research – including approaches capable of taking seriously both the need for valid consent and the fact that the specific details of future research to be undertaken on collected samples and data cannot be known at the time of initial recruitment. The most prominent amongst these is what is known as ‘broad consent’, a model of consent that can allow the use of biological samples and associated data both in specific immediate research and in future research, subject to appropriate governance arrangements. Proponents of broad consent have argued that this model of consent is legitimate because it is consistent with current practices, it respects the autonomy of participants, and the risks involved are minimal (Fisher & Layman, 2018; Hansson et al., 2006; Helgesson, 2012; Kaye, 2010; Petrini, 2010; Sheehan, 2011; Staunton et al., 2019). Their argument is that insisting on very specific informed consent would fail to take seriously the autonomy of those who wish to give their broad consent and would make much important research non-viable. It would mean, for example, returning to research participants for re-consent for each new research use of their data. This, it is argued, presents significant practical challenges and costs which could together undermine important research that participants would likely want to see take place. The conditions for legitimising broad consent are that personal details are handled safely, ‘future’ research is reviewed and approved by an Institutional Review Board (IRB) or Research Ethics Committee (REC) or by a data and biospecimen access committee with appropriate representation, and participants are given the opportunity to withdraw at any time. However, critics of broad consent have argued that it is misleading to ask for participants’ informed consent for research that is unforeseen and has not been specified in a research protocol. For these critics, the only form of valid consent is consent for each very specific research activity.

Public views and perceptions of research with stored samples and biobanking research are increasingly being reported in high-income countries such as the United States of America and the United Kingdom and are contributing to the development of regulatory frameworks (Garrison et al., 2016; Gibbons & Kaye, 2007; IPSOS Mori, 2019; Pace et al., 2006; Steinsbekk et al., 2013; Wendler & Emanuel, 2002). Much less work of this kind has been undertaken on the African continent, despite the fact that recent years have seen a rapid growth in genomic research in many African countries as a result of initiatives such as H3Africa. Some empirical studies in Africa have reported that although there is general support for genomic studies, particularly on the reuse of samples, there are also concerns about how the interests of key stakeholders such as participants (i.e. sample donors) and the broader local communities from which samples are collected can and should be ascertained and protected. For example, the findings from a study by Moodley and Singh (2016) suggest that although research participants in the Western Cape in South Africa support broad consent for reuse of samples, the majority of the participants expressed a desire to be informed about what future studies will be conducted on their samples. Igbe and Adebamowo (2012) reported that participants in Nigeria were generally supportive of sample storage and future uses on condition that procedures were in place to prevent the unethical use of the specimens collected. In this collection of three papers, we contribute to this growing body of work to report the views of research participants and other key stakeholders on sample storage and the securing of consent in genomic research in Zambia, Uganda, and Ghana (Mweemba et al., 2019; Rutakumwa et al., 2019; Tindana et al. 2020).

The focus of the paper by Mweemba et al. reports on the findings of a qualitative study with participants recruited for an H3Africa study on rheumatic heart disease in Zambia. The study, which focused on participants’ views on broad consent, sample and data sharing, and secondary use studies, found that broad consent tended to be viewed as an acceptable approach to consent for genomic research as long as the reasons for storing samples and data for future research were clearly explained at the time of initial consent. Whilst accepting the idea of broad consent, a subset of interviewees took the view that some limitations on future uses might be appropriate by, for example, limiting such uses to research into the same disease as that which motivated original recruitment. The headline finding of the paper is that broad consent is an appropriate model for genomic research in Africa as long as appropriate governance is in place overseeing the limits of future research use.

The focus of the paper by Rutakumwa et al., is broader in focus exploring the view of Ugandan research participants about the requirements for ethical genomic research in Uganda. The study suggests that conventional ethics standards and practices may not be sufficiently responsive to participant and community concerns and interests in addressing the ethical complexity of the ethical challenges in research. The findings reported in the paper suggest three areas in which work is required to improve the responsiveness of genomic research in this regard. These are: the importance of deemphasising the roles of experts and institutions in consent processes, achieving greater clarity about the timing and nature of the feedback of health-related findings and updates on project progress, and more effective support for research participants during and after the study.

Finally, the paper by Tindana et al., reports on interviews with key stakeholders in Ghana on consent practices in relation both to the genomics research and to the broader practice of biobanking. The study included interviews with genomics researchers, fieldworkers, laboratory staff, and members of ethics committees, in addition to both participants and those who refused to participate. The paper reports a willingness to participate in research initiatives involving the collection and sharing of samples and data for future research, as long as there are good governance structures in place and, importantly, that participants receive feedback about the progress of the research endeavour as a whole, and about the research uses of their samples and data. The study findings emphasise the importance of the discussion of feedback plans at the time of initial consent.

Taken together, these three papers make an important contribution to debates on the ethics of genomic research in Africa at a time in which the scale of such research is rapidly increasing in scale, scope, and importance.
